# RNAseq analysis reveals pathways and candidate genes associated with salinity tolerance in a spaceflight-induced wheat mutant

**DOI:** 10.1038/s41598-017-03024-0

**Published:** 2017-06-02

**Authors:** Hongchun Xiong, Huijun Guo, Yongdun Xie, Linshu Zhao, Jiayu Gu, Shirong Zhao, Junhui Li, Luxiang Liu

**Affiliations:** Institute of Crop Science, Chinese Academy of Agricultural Sciences/National Key Facility for Crop Gene Resources and Genetic Improvement, National Center of Space Mutagenesis for Crop Improvement, Beijing, 100081 China

## Abstract

Salinity stress has become an increasing threat to food security worldwide and elucidation of the mechanism for salinity tolerance is of great significance. Induced mutation, especially spaceflight mutagenesis, is one important method for crop breeding. In this study, we show that a spaceflight-induced wheat mutant, named *salinity tolerance 1* (*st1*), is a salinity-tolerant line. We report the characteristics of transcriptomic sequence variation induced by spaceflight, and show that mutations in genes associated with sodium ion transport may directly contribute to salinity tolerance in *st1*. Furthermore, GO and KEGG enrichment analysis of differentially expressed genes (DEGs) between salinity-treated *st1* and wild type suggested that the homeostasis of oxidation-reduction process is important for salt tolerance in *st1*. Through KEGG pathway analysis, “Butanoate metabolism” was identified as a new pathway for salinity responses. Additionally, key genes for salinity tolerance, such as genes encoding arginine decarboxylase, polyamine oxidase, hormones-related, were not only salt-induced in *st1* but also showed higher expression in salt-treated *st1* compared with salt-treated WT, indicating that these genes may play important roles in salinity tolerance in *st1*. This study presents valuable genetic resources for studies on transcriptome variation caused by induced mutation and the identification of salt tolerance genes in crops.

## Introduction

Soil salinity is a major abiotic stress that causes substantial losses in productivity in world agriculture^1^. Over 6% of the world’s total land area is affected by excessive salinity, and this proportion is increasing in irrigated agricultural areas and in semi-arid areas^2–4^. However, despite the very real threat it represents, our current understanding of the molecular mechanisms underlying salinity tolerance in crop plants remains very limited. Bread wheat (*Triticum aestivum* L.) is one of the most important food crops in the world^5^, but wheat yields are seriously threatened by salinization^6, 7^. Therefore, both the development of salt-tolerant wheat materials and the elucidation of the mechanisms of salinity resistance are of great significance.

In plants, several metabolic processes and key genes have been reported to be strongly correlated with salinity stress. It has been reported that Sulphur (S) assimilation plays a pivotal role in the metabolic modifications that occur under salt stress, and increased S levels have been shown to increase salt tolerance in plants^8, 9^. S-adenosyl methionine is the precursor of polyamines (PAs), which are closely associated with plant resistance to salinity stress^10–13^. The major PAs found in plants are putrescine (Put), spermidine (Spd), and spermine (Spm). Put can be synthesized from L-arginine by arginine decarboxylase (ADC) or ornithine decarboxylase (ODC)^11^. Polyamine oxidases (PAOs) catalyze the back conversion of PAs to Spd or Put and help maintain PA homeostasis^14^. It has been suggested that PAOs function in stress resistance by mediating PA homeostasis^15, 16^.

In *Arabidopsis*, several *Salt Overly Sensitive* (*SOS*) genes have been identified. *SOS1* is a plasma membrane Na^+^/H^+^ antiporter that is responsible for the exclusion of sodium from the cytosol^17^. The serine/threonine type protein kinase SOS2 (CIPK24) interacts with the calcium sensor SOS3 (CBL4) to regulate the Na^+^/H^+^ exchanger *SOS1*, thereby conferring salt resistance^17–20^. It has been suggested that in the tree, poplar, CIPK24, CIPK25 and CIPK26 interact with CBL1 to regulate Na^+^/K^+^ homeostasis^21^. Additionally, the *CIPK* genes from wild barley and maize also have been implicated in salt tolerance responses^22, 23^.

With the development of high throughout sequencing technologies, transcriptome analyses in plants exposed to salinity stress have been reported for common bean^24^, Kentucky bluegrass^25^, ice plant^26^, canola^27^, desert poplar^28^, and Kharchia local wheat^29^. These studies have suggested that differentially expressed genes relating to oxidation-reduction processes and proline metabolism, as well as many transcription factors, all likely play roles in salt tolerance^25, 27^. In a recent study transcriptome-level analysis was used to examine the root responses to salinity in an Indian wheat salt-tolerant variety^29^. However, only limited information is available regarding differences in shoot transcriptomes between salt-tolerant and susceptible wheat.

Spaceflight breeding has for a long time been an important source for mutation breeding, and many cultivars have been released via this programme^30^. Despite these successes, there have been few reports of transcriptome analyses seeking to characterize transcriptomic sequence variation resulting from a space-induced mutation. Previous study suggested that spaceflight-induced mutation affects the growth and development of wheat plants at the physiological level^31^. Several studies have focused on gene expression variation induced by spaceflight^32–36^ but very few studies have reported transcriptomic sequence variation induced by spaceflight. In this study, we report the identification of a salt-tolerant wheat mutant *st1* derived from spaceflight mutagenesis and the characteristics of transcriptomic sequence variation induced by spaceflight were analyzed in detail, providing novel information about sequence variation induced by the spaceflight environment. The gene expression changes between *st1* and wild type (WT) plants in response to high salt treatment were also analyzed, and putative candidate genes associated with salt tolerance in *st1* are discussed. This study serves as a foundation for studies seeking to characterize transcriptome sequence variation induced by spaceflight and serves as a resource for researchers seeking to identify key genes involved in salinity tolerance in wheat.

## Results and Discussion

### A spaceflight-induced mutant in wheat confers tolerance to high salinity

**Figure 1:**
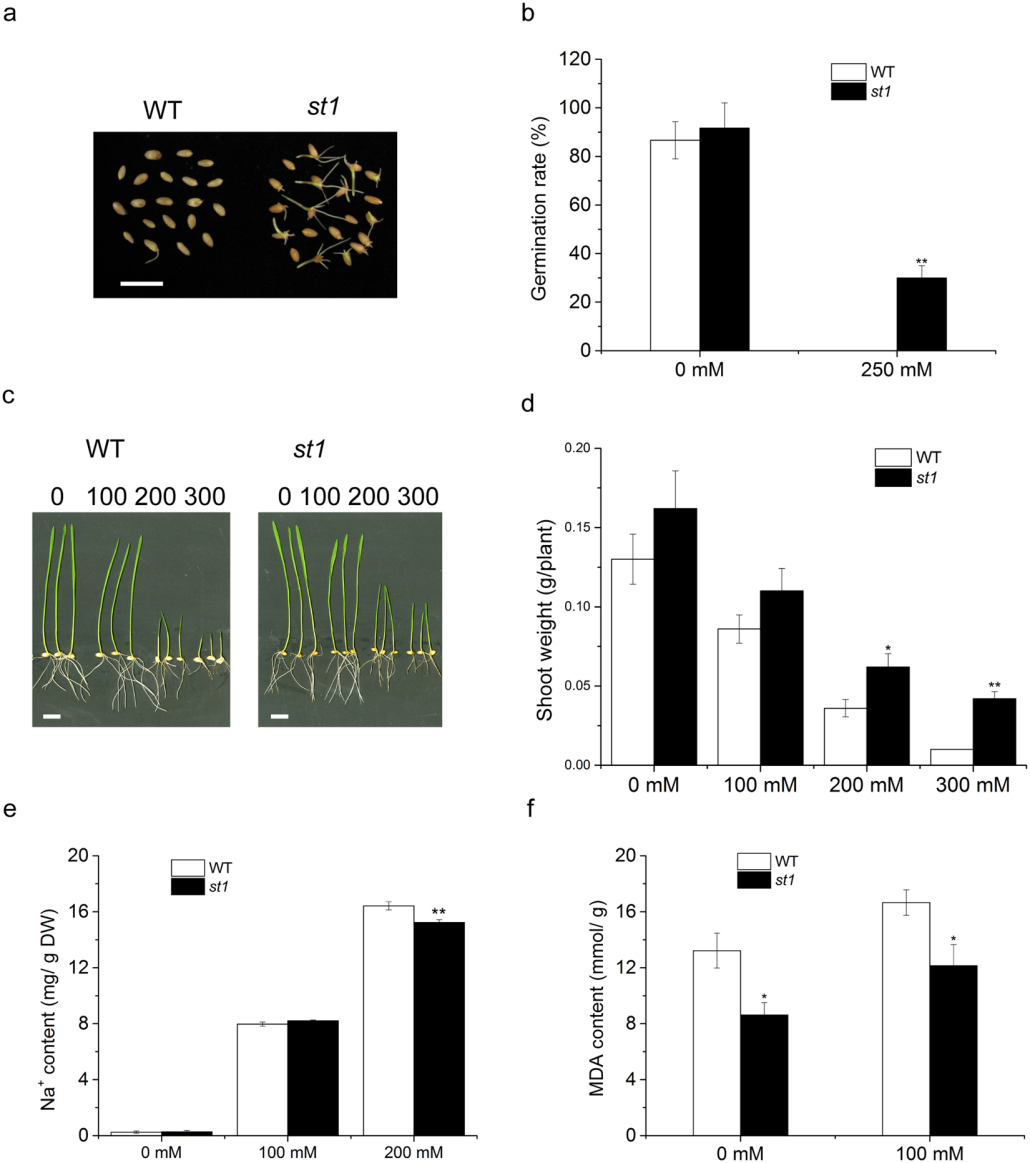
Phenotypical comparisons of WT and mutant in response to salt treatment. (**a**) The phenotypes and (**b**) germination rate of WT and *st1* seeds exposed to 250 mM NaCl. (**c**) The phenotypes and (**d**) shoot weights of WT and *st1* plants grown in 0, 100, 200, or 300 mM NaCl after germination. Bars = 2 cm. (**e**) The Na^+^ concentration in the shoots of WT and *st1* plants treated with 100 or 200 mM NaCl or without treatment. (**f**) The MDA content in the shoots of WT and *st1* plants treated with 100 mM NaCl. Values are means ± SD. Student’s t-tests were used to assess the significance of differences from WT plants, *P < 0.05, **P < 0.01.

We identified a salinity-tolerant mutant *st1* in a screen of a large number of induced wheat mutants grown in hydroponics with high salinity. The germination rate of *st1* was significantly higher than that of WT when assayed on 250 mM NaCl (Fig. 1a,b). Moreover, *st1* seedlings clearly grew better than WT when plants were exposed to high concentrations of NaCl (Fig. 1c). Mutant plants treated with 200 or 300 mM NaCl had significantly increased shoot weight compared to WT (Fig. 1d). With 200 mM NaCl treatment, the sodium concentration in the shoot was notably decreased in *st1* compared to WT (Fig. 1e). Additionally, the malondialdehyde (MDA) content, which is typically held to reflect the intensity of damage to the plasma membrane under stress, was significantly lower in the leaves of *st1* than in the leaves of WT (Fig. 1f). All of these results indicate that the spaceflight-induced wheat mutant *st1* plants were more tolerant to salt stress than were WT plants.

### RNAseq analysis of WT and mutants shoot under salinity stress

The transcript changes in WT and the *st1* mutant with or without salinity treatment were investigated using RNAseq analysis based on high-throughput sequencing. Sequencing of each genotype/treatment group was done independently twice, and each sample was pooled from five plants; thus, 8 cDNA libraries were constructed. The raw transcriptome datas generated from this study have been submitted to the Sequence Read Archive (SRA), National Centre for Biotechnology Information (NCBI) with the accession number SRR5277542. The number of raw reads from each library ranged from 43 million to 65 million. After removing poor quality reads, adaptor contamination, and low quality regions, between 42 to 63 million clean reads were obtained from each of the libraries. From each library >67% of reads mapped to the release-31 version of wheat reference genome, including >58% with unique mapping and <9% multiple mapping (Supplementary Table S1). Furthermore, >98% and >95% of the clean reads had quality scores at the Q20 or Q30 (an error probability for base calling of 1% or 0.1%)^37^ level, respectively. The density of reads was equally distributed across all chromosomes of the wheat reference genome (Supplementary Fig. S1) and 70% of the genes in the wheat genome database were detected within these samples, suggesting the RNAseq datas are well covered the wheat transcriptional region. Additionally, there was a clear linear relationship between the gene expression levels of the two biological replicates for each genotype/treatment group (R^2^ > 0.97, Supplementary Fig. S2).

**Figure 2:**
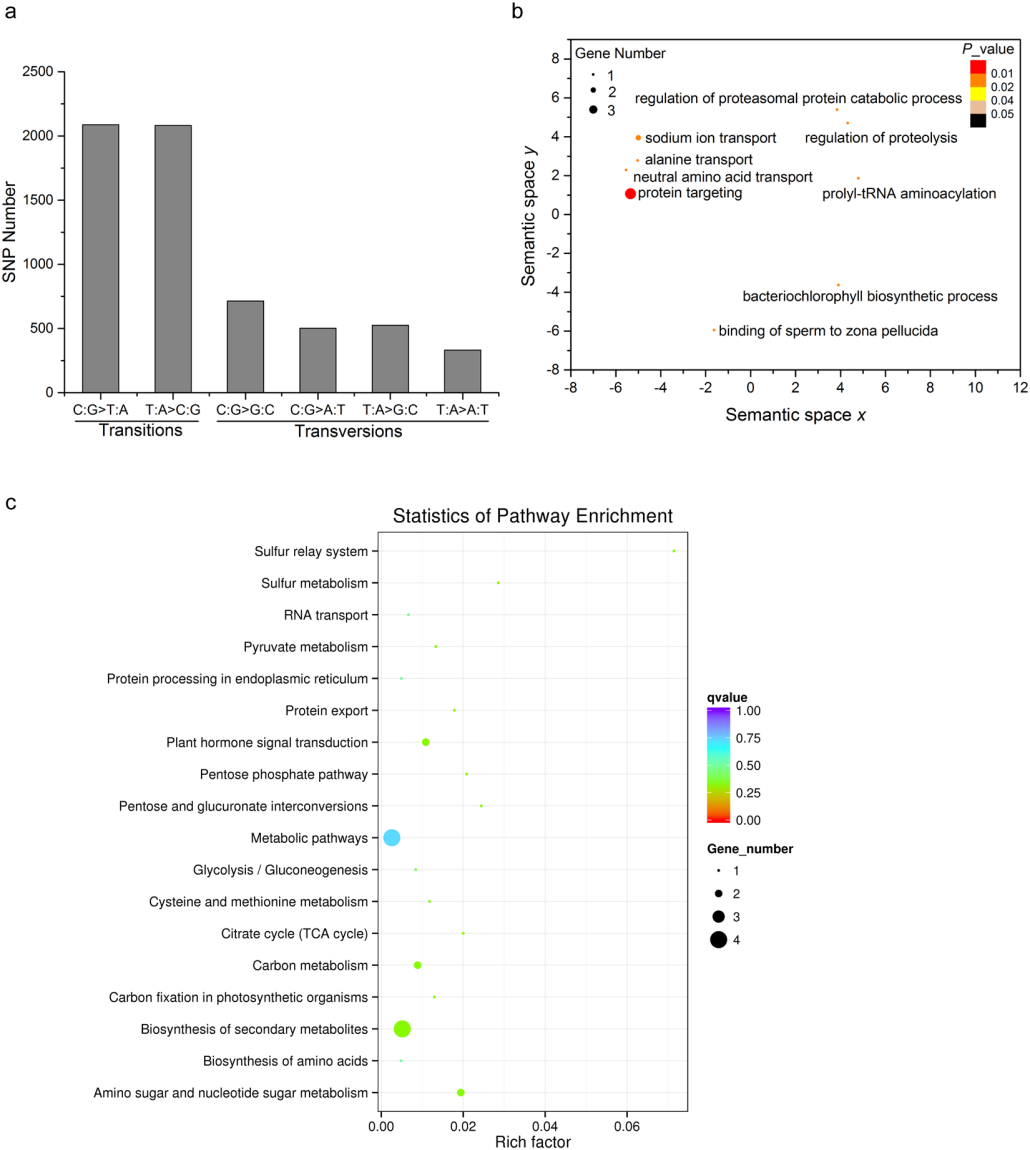
The characteristics of SNPs and GO terms and KEGG pathways enriched in *st1* mutated genes. (**a**) Numbers of specific types of SNPs in the transcriptome of the spaceflight-induced mutant *st1*; (**b**) The top ten GO terms enriched in genes with termination and missense mutations determined based on the lowest over-represented *p* values were analyzed by REVIGO. Circles in closer proximity have GO terms that are more closely related. The size of the circle indicates the number of mutated genes. The color of the circle represents the statistical significance of the enriched GO terms based on the over-represented *p* value; (**c**) The KEGG pathways enriched in genes with termination and missense mutations. The rich factor reflects the proportion of mutated genes in a given pathway. The number of mutated genes in the pathway is indicated by the circle area, and the circle color represents the ranges of the corrected *P* value.

Among the homozygous variants, 6211 SNPs and 414 InDels, distributed among 21 chromosomes, were detected (Table 1). This is consistent with the findings of a study of transcriptome sequence variation in a space-induced mutant of Kentucky bluegrass, where more SNPs than InDels were also identified^38^. Of the 6211 SNPs identified in our study, there were more transitions than transversions with a transitions: transversions (Ti: Tv) ratio of 2.0 (Fig. 2a). High Ti: Tv values have also been reported for other types of mutagenesis like fast-neutron irradiation^39^ and EMS mutation^40^. However, the distribution of transitions and transversions is roughly similar in gamma-ray-induced mutation^40^. Among the four possible types of transversions, the number of C/G to G/C transversions was the highest, accounting for 34.4% of point mutations, while the number of T/A to A/T transversions was the lowest, accounting for 16.0% (Fig. 2a). In contrast, it has been reported that EMS-, gamma-ray-, and fast-neutron- induced mutations exhibit lower numbers of C/G to G/C transversions compared with other types of transversions^39, 40^.

**Table 1:** The number of SNPs and InDels between WT and *st1* found on each of the 21 wheat chromosomes.

	1A	1B	1D	2A	2B	2D	3A	3B	3D	4A	4B	4D	5A	5B	5D	6A	6B	6D	7A	7B	7D	Totally
SNPs	173	643	13	113	450	72	215	1422	60	277	237	22	594	401	188	73	670	54	148	190	196	6211
InDels	8	28	0	10	28	5	25	115	4	18	22	1	48	22	17	1	35	4	3	10	10	414

We next looked at the SNPs located within genes. In six genes SNPs resulted in termination mutations, and 110 genes of SNPs resulted in missense mutations. Among the genes with termination and missense mutations, only seven genes had increased expression and four genes had decreased expression (fold change ≥2) in the *st1* compared to WT (Supplementary Table S2). Furthermore, the enriched GO terms and KEGG pathways for genes with termination and missense mutations were determined (Supplementary Table S2 and Fig. 2b,c). REVIGO was used to summarize the enriched GO terms (http://revigo.irb.hr/). The top ten biological GO terms in biological process enriched in genes with termination and missense mutations, defined based on the lowest over-represented *p* values, were analyzed by REVIGO (Fig. 2b). This program removes redundant GO terms and the similarity between terms is reflected by semantic space^41^. Interestingly, two mutated genes are annotated with the enriched GO term “sodium ion transport” (Fig. 2b). It has been reported that the Na^+^/H^+^ antiporter SOS1 is important for the exclusion of sodium in *Arabidopsis* root cells^17^, and the wheat sodium transporters HKT1; 5A^42^ and HKT1; 5D^43^ function in decreasing Na^+^ concentration in the leaves and thus improve salt tolerance. These mutated genes associated with sodium ion transport offer important clues for the mechanism of salt tolerance in the *st1* mutant plants, and their gene functions need to be further analyzed. KEGG pathway analysis indicated that some important pathways, such as “metabolic pathways”, “carbon metabolism” and “plant hormone signal transduction”, were enriched in mutated genes (Fig. 2c, Supplementary Table S2), suggesting that spaced-induced mutation of metabolism genes led to variation in metabolism and changes the characteristics of the mutant.

**Figure 3:**
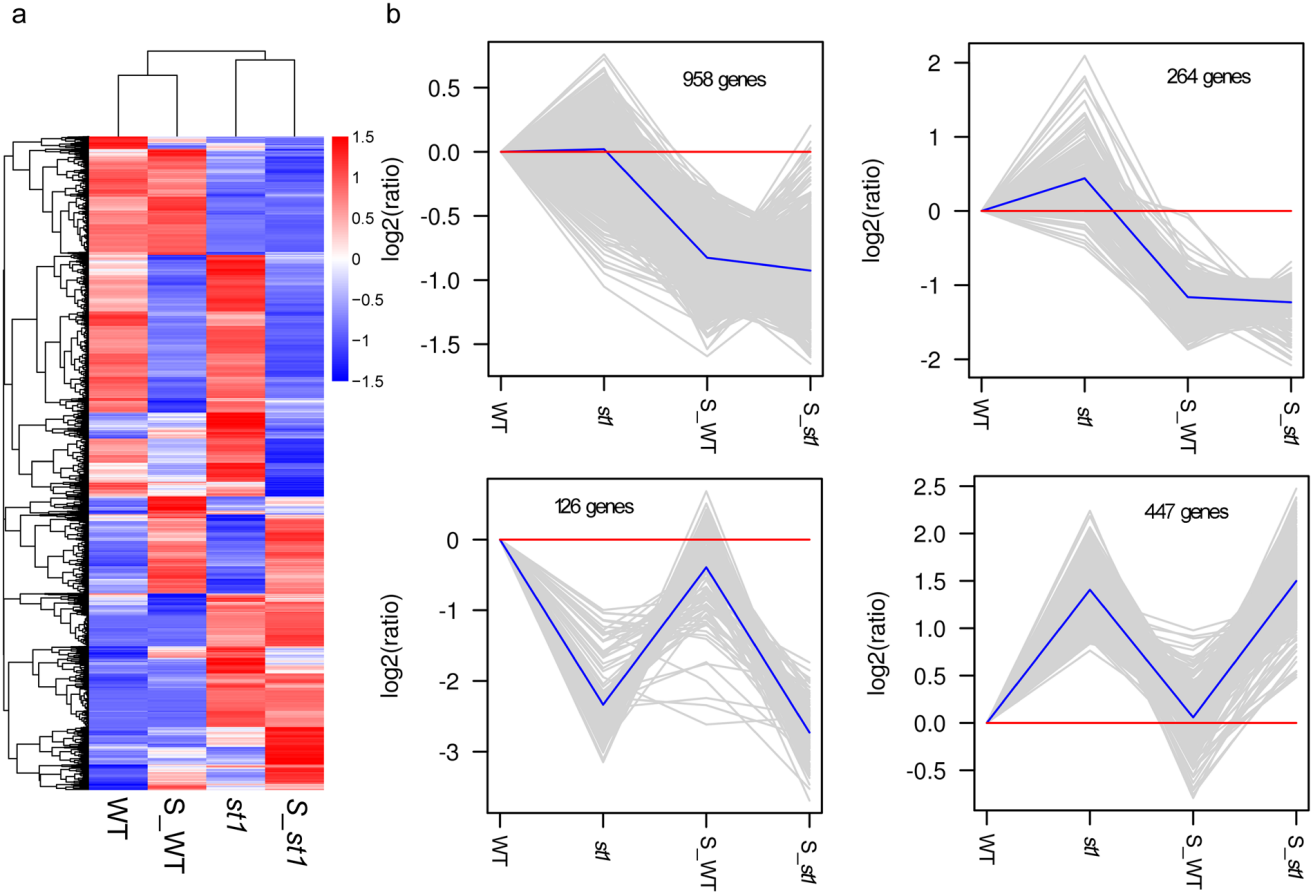
Clustering of DEGs between WT and *st1* in response to salt treatment. (**a**) Hierarchical clustering of all DEGs; (**b**) The top four subclusters from the hierarchical clustering in (**a**) based on numbers of DEGs. Gray lines show the relative expression levels of DEGs in the subcluster in WT and *st1* with or without salt treatment. Blue lines show the average values for relative expression in each subcluster. The different samples are shown on the x-axis and the y-axis indicates the relative expression level.

**Figure 4:**
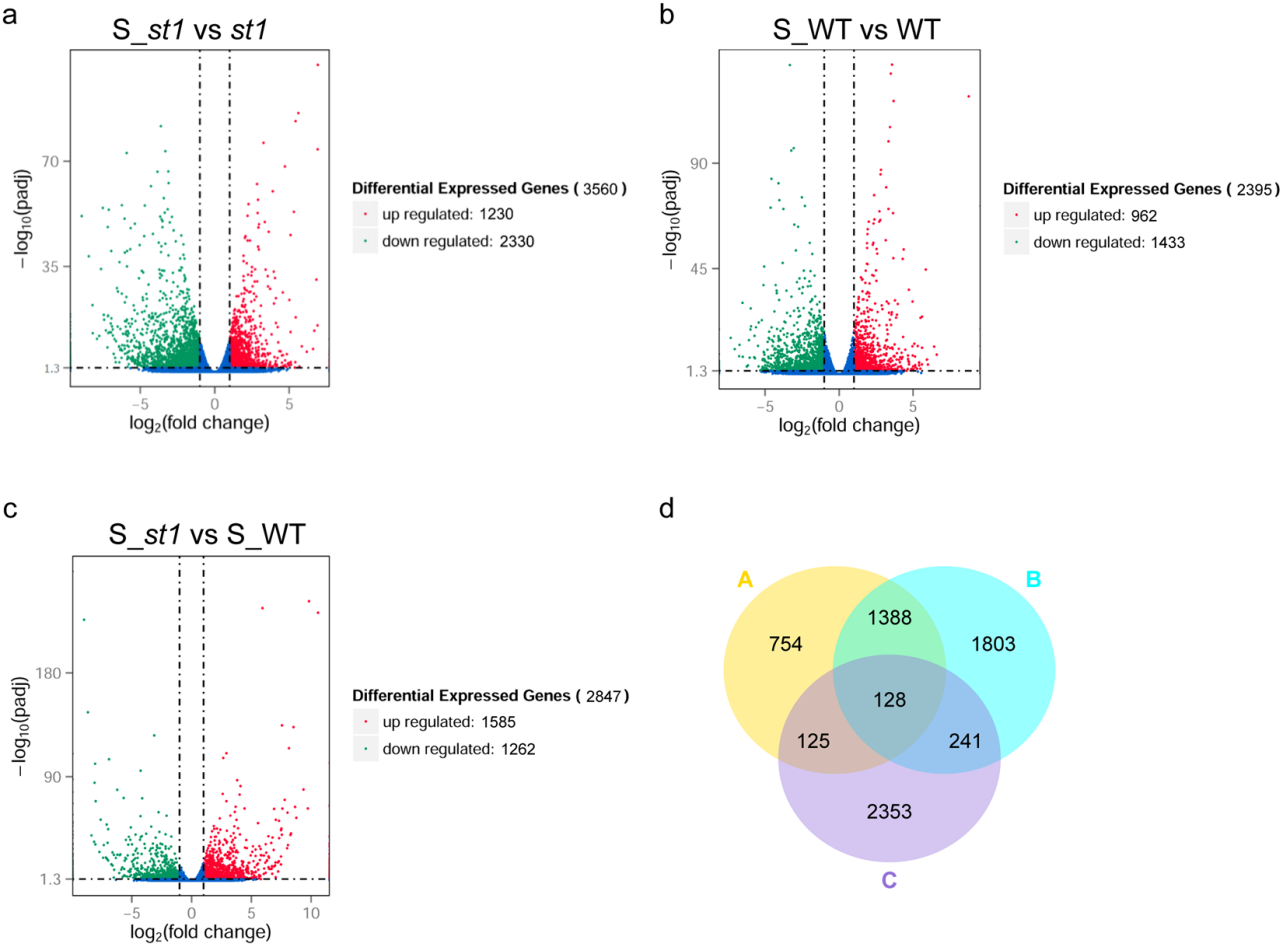
Volcano plots and Venn diagrams of DEGs. (**a**–**c**) DEGs between salt-treated *st1* and *st1* (S_ *st1* vs *st1*, **a**), salt-treated WT and WT (S_WT vs WT, **b**), salt-treated *st1* and salt-treated WT (S_ *st1* vs S_WT, **c**) displayed by volcano plots. The abscissa shows the fold change difference in the expression of genes in different comparison groups, and the vertical coordinates indicate the adjusted *P*-values for the differences in expression. Genes without significant differences are indicated by blue dots. The up-regulated genes are represented by red dots, and the down-regulated genes are represented by green dots. (**d**) Venn diagrams illustrating the overlap in DEGs between WT and *st1* in response to salinity stress. The numbers in each circle (Circle A, S_WT vs WT; Circle B, S_ *st1* vs *st1*; Circle C, S_ *st1* vs S_WT) indicate the total number of different genes in each comparison group, and the number in the overlapping areas is the number of shared genes between two comparison groups.

Analysis of differentially expressed genes (DEGs) was another way to identify genes that may be responsible for salinity tolerance in the *st1* mutant. Therefore, DEGs were identified from the RNA-seq data based on the criteria of fold change ≥2 for the comparisons salinity (S)_*st1* vs *st1*, S_WT vs WT, S_*st1* vs S_WT and an FDR < 0.05 (Supplementary Table S3). Cluster analysis of DEGs suggested that the number of genes down-regulated by salinity in both WT and *st1* was generally higher compared with other subclusters (Fig. 3). In the S_*st1* vs *st1* comparison group, 3560 DEGs, including 1230 with up-regulated expression and 2330 with down-regulated expression, were identified (Fig. 4a). And 2395 transcripts (962 up-regulated and 1433 down-regulated genes) showed significant changes in S_WT vs WT (Fig. 4b). In S_*st1* vs S_WT, 1585 genes were up-regulated and 1262 genes were down-regulated (Fig. 4c). Venn diagrams indicate that a large proportion of DEGs (63.3%) in S_WT vs WT overlap with the S_*st1* vs *st1* DEGs. However, only a small proportion of DEGs (17.4%) in the S_*st1* vs S_WT comparison overlap with the salinity responsive genes (i.e. S_*st1* vs *st1* or S_WT vs WT DEGs). Because *st1* is more tolerant to salinity compared with WT, the salt responsive genes of *st1* that are also differentially expressed in the S_*st1* vs S_WT comparison (in total 369 genes, Fig. 4d) are more likely to play roles in the salinity tolerance of *st1*.

### Functional categorization of salinity-treated WT and *st1* mutant DEGs

**Figure 5:**
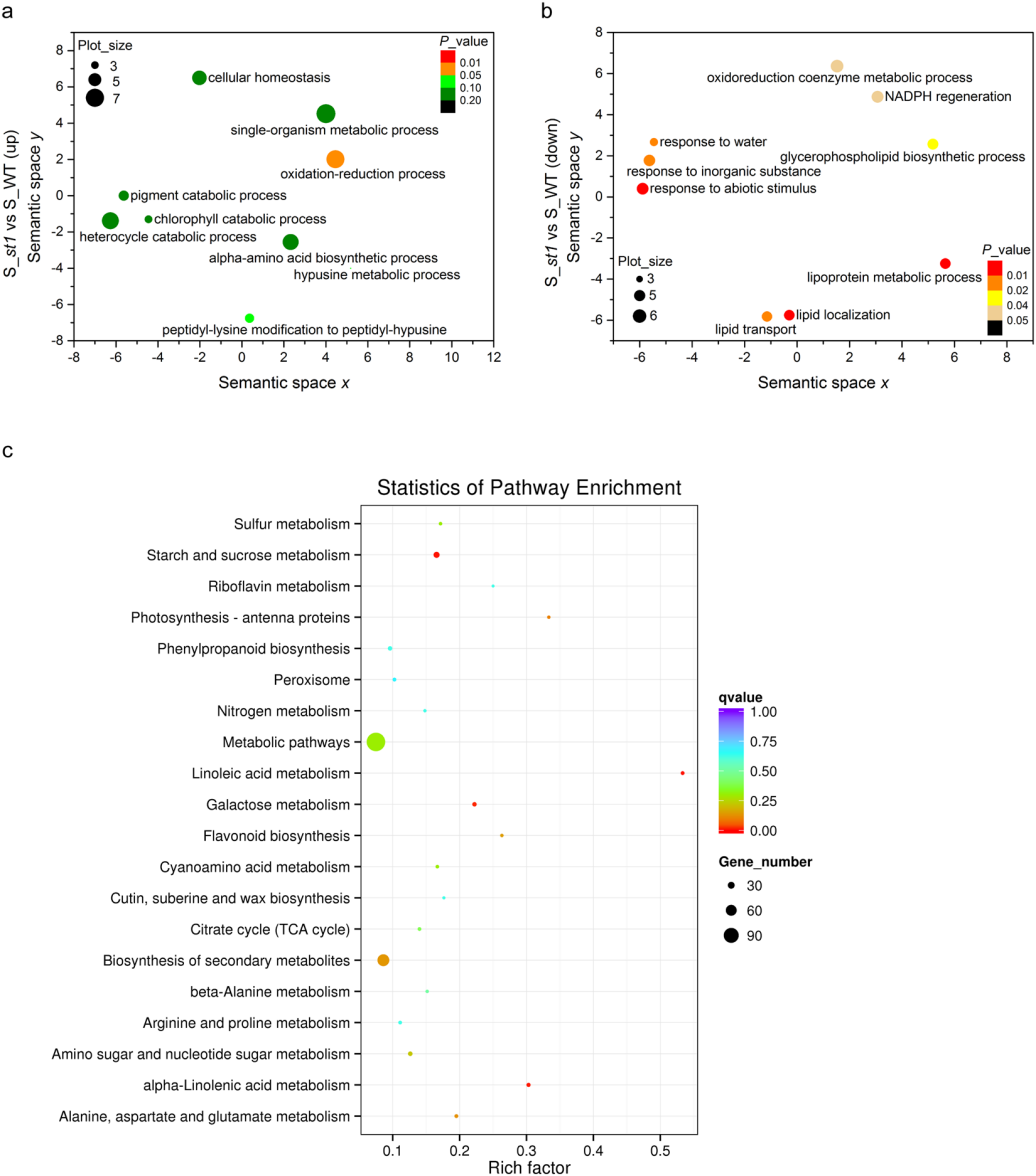
GO enrichment and KEGG pathway analysis. The top ten GO terms enriched in S_ *st1* vs S_WT DEGs, determined based on the lowest *p* values, were analyzed by REVIGO. (**a**,**b**) The enriched GO terms from the REVIGO analysis for up-regulated (**a**) and down-regulated (**b**) genes in the S_ *st1* vs S_WT comparison group. Circles in closer proximity have more closely related GO terms. The size of the circles indicates the number of child GO terms. The color of the circle represents the significance of the enriched GO terms. (**c**) KEGG pathways enriched in S_ *st1* vs S_WT up-regulated genes. The abscissa represents the richness factor reflecting the proportion of DEGs in a given pathway. The number of DEGs in the pathway is indicated by the circle area, and the circle color represents the range of the corrected *P* values.

The top ten GO terms in biological process category enriched in DEGs of the S_*st1* vs S_WT group based on the lowest *p* values were analyzed by REVIGO. For the up-regulated genes, “oxidation-reduction process” was the only significantly enriched GO term (*p* < 0.05) (Fig. 5a), suggesting the importance of this process for salinity resistance in *st1*. Consistent with this finding, previous transcriptome profiling experiments have indicated that oxidation-reduction processes are related to salt tolerance in various plants^25, 27, 28^. In contrast, in the S_*st1* vs S_WT comparison, enriched highly significant terms (*p* < 0.05) for down-regulated DEGs included “response to water”, “response to inorganic substance”, “response to abiotic stimulus”, “lipoprotein metabolic process”, “lipid transport”, “lipid localization”, “oxidoreduction coenzyme metabolic process”, “NADPH regeneration” and “glycerophospholipid biosynthetic process” (Fig. 5b). The fact that genes related to these processes, especially those involved in response to water, inorganic substance and abiotic stimulus, were up-regulated in the salt-treated WT compared to the salt-treated mutant, indicates that the WT is more sensitive than *st1* to high salt, which is consistent with its salt-sensitive phenotype (Fig. 1). Sixty-six GO biological process terms were significantly enriched in the S_*st1* vs *st1* up-regulated genes, whereas only 11 GO terms were enriched in S_WT vs WT up-regulated genes (Supplementary Table S4). Furthermore, the number of up-regulated genes involved in “response to water stimulus”, “response to abiotic stimulus” and “response to chemical stimulus” was generally higher for the S_WT vs WT comparison compared with the S_*st1* vs *st1* comparison (Supplementary Table S4), indirectly suggesting the greater sensitivity of WT to salinity compared with *st1*. It is possible that the up-regulation of multiple genes involved in various biological processes in *st1* under salinity contributes to salinity tolerance.

KEGG pathway enrichment analysis showed that, among the top 20 enriched pathways (based on corrected *p*-value) for the S_*st1* vs S_WT up-regulated genes, the “Starch and sucrose metabolism”, “Linoleic acid metabolism”, “Galactose metabolism” and “alpha-Linolenic acid metabolism” pathways were significantly enriched (Fig. 5c). These pathways have been well-documented to play roles in salinity tolerance^3, 44, 45^. For the down-regulated genes in this comparison, no pathway was significantly enriched, but the “Biosynthesis of secondary metabolites” pathway had the largest number of genes (Supplementary Fig. S3 and Supplementary Table S5). The pathways involved in “Arginine and proline metabolism”, “Alanine, aspartate and glutamate metabolism”, “Linoleic acid metabolism”, “Plant hormone signal transduction”, “Citrate cycle (TCA cycle)”, “Pyruvate metabolism”, “Biosynthesis of secondary metabolites”, and “Butanoate metabolism” were commonly significantly enriched (*p* < 0.05) in S_*st1* vs *st1* and S_WT vs WT up-regulated genes (Supplementary Table S5). This suggests that these pathways are active under salinity stress. Among these pathways, the “Butanoate metabolism” pathway has previously not been shown to function in salinity response. 4-amino-butanoate is one of the intermediate products of butanoate metabolism, and it is closely connected with the PA pathway, which is suggested to be involved in plant resistance to salinity stress^10–13^. Additionally, “Butanoate metabolism” is also closely associated with the salinity responsive “Alanine, aspartate and glutamate metabolism” and “TCA cycle” pathways^27^. Therefore, the expression of genes involved in butanoate metabolism was affected by salinity stress in *st1* and WT.

### Key genes responsible for salt tolerance in the *st1* mutant

**Figure 6:**
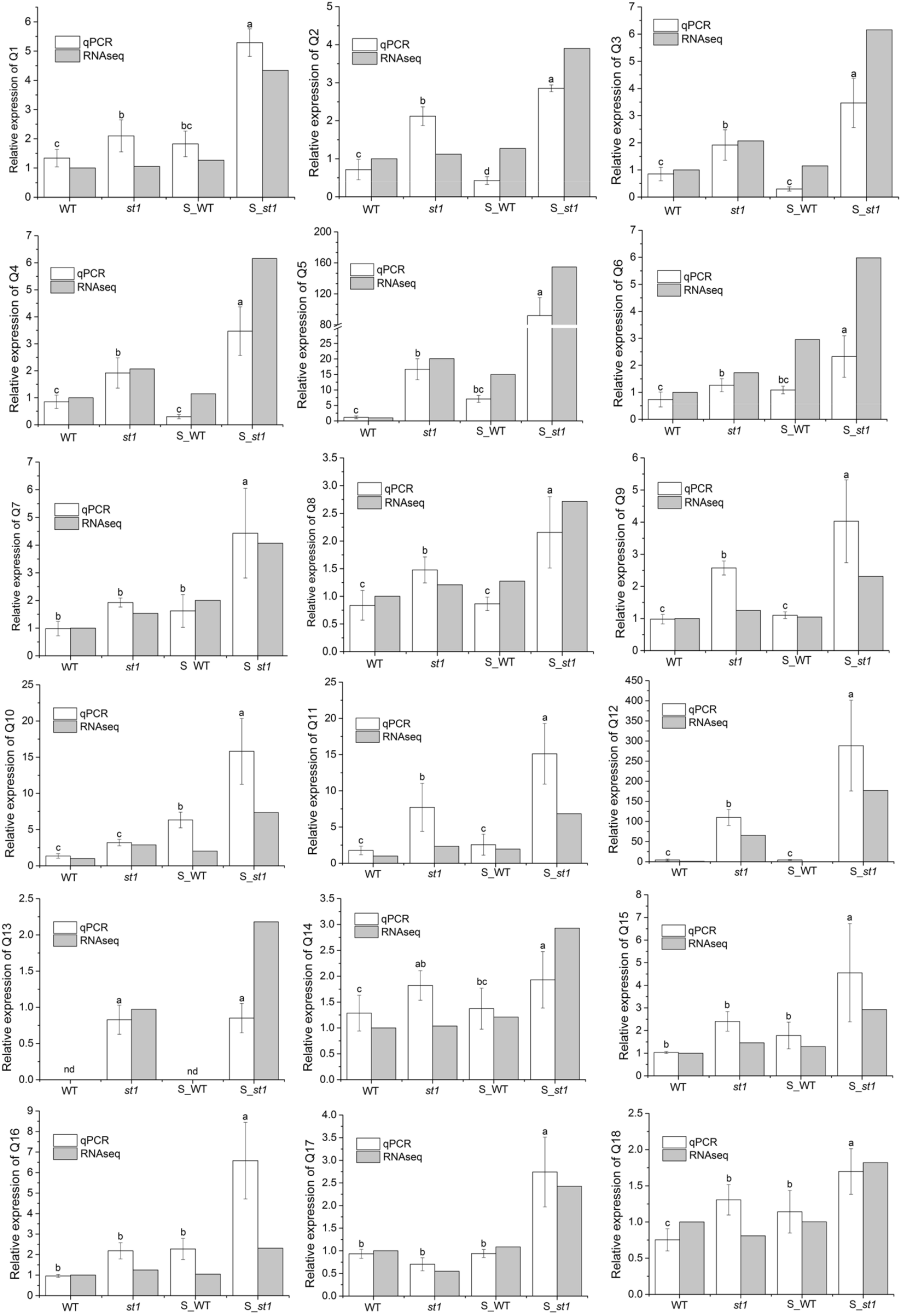
Expression patterns of selected genes in WT and *st1* with and without salt treatment determined by RNA-seq and qPCR. The gene numbers (Q1–Q18) correspond to the gene labels in Table 2. The RNA-seq values represent the ratio of the expression level in *st1* to the expression level in WT. The expression level for each sample is the average of two replicates. For qPCR, the data are means ± SD from three independent replicates, and different letters indicate significant differences between samples at *P* < 0.05 based on SAS statistic analysis.

Since the salt responsive genes showing differential expression in the S_*st1* vs S_WT comparison might be important for salinity tolerance in *st1*, the overlap between DEGs from the S_*st1* vs *st1* and S_*st1* vs S_WT comparisons were analyzed in detail (Table 2, Supplementary Table S6), especially the up-regulated genes. We also confirmed the RNA-seq results for selected DEGs by qPCR (Fig. 6). Among the up-regulated genes, most genes were involved in arginine and proline metabolism, oxidoreductase, hormone metabolism, transcription factor and signaling regulation (Table 2). Additionally, genes involved in “Alanine, aspartate and glutamate metabolism”, “S assimilation and metabolism”, “Glutathione metabolism”, “Flavonoid metabolism”, “disease related” and “energy metabolism”, were also up-regulated in both the S_*st1* vs *st1* and the S_*st1* vs S_WT comparisons (Supplementary Table S6). Generally, distinct genes were observed in the down-regulated group (Supplementary Table S6).

**Table 2:** List of putative candidate genes for salt tolerance in *st1*.

Gene_id	Gene description	S_*st1* vs *st1* ^a^	S_*st1* vs S_WT^a^
**Arginine and proline metabolism**			
Traes_7AL_113B64D31 (Q1)	polyamine oxidase	2.2	1.9
Traes_7AL_425787F27 (Q2)	polyamine oxidase	1.9	1.8
Novel06546 (Q3)	polyamine oxidase	1.8	2.7
Traes_2BL_DA615F345 (Q4)	probable polyamine oxidase 2	1.6	1.0
Novel06385	polyamine oxidase	2.0	2.5
Traes_1DS_62B0EC35F	arginine decarboxylase	3.4	1.5
**Oxidation-reduction**			
Traes_3AS_D6DD0F6B9 (Q5)	cytochrome P450 734A6-like	3.1	3.5
Traes_2DS_21F32D71E (Q6)	cytochrome P450 76M5-like	1.9	1.2
Traes_5BL_175E0D966 (Q7)	cytochrome P450 76M5-like	1.5	1.2
Traes_2AL_01B85FEE0 (Q8)	cytochrome P450 87A3-like	1.3	1.3
Traes_5BL_A5F97A71F (Q9)	cytochrome P450	1.0	1.3
Traes_6AS_DE31C9DA8	cytochrome P450 76M5-like	1.5	4.5
TRAES3BF021100050CFD_g	cytochrome P450	1.1	2.3
Novel05282	cytochrome P450	2.3	2.5
Traes_4AS_E4D61C2E6	cytochrome P450 704B1	1.6	1.0
Novel03868	cytochrome P450	4.3	1.2
Traes_6AS_F061F607E	cytochrome P450-like	2.8	1.4
Traes_7DL_4851726D0	L-ascorbate oxidase-like	3.2	2.5
Novel08581	ascorbate-dependent oxidoreductase	1.4	2.0
**Hormone related**			
Traes_4DL_B3E978E9F (Q10)	allene oxide synthase (TaAOS)	1.5	2.0
Traes_6DL_94DCF0B70	putative 12-oxophytodienoate reductase 11	3.1	2.5
Traes_2BL_0D4EA8B58	12-oxophytodienoic acid reductase 2	1.6	1.0
Traes_6BS_B26FD03C8	lipoxygenase	1.3	1.2
Traes_4BS_71CB57A0D	lipoxygenase-1 (Lpx-B1.1)	1.0	2.9
Novel05745	lipoxygenase	3.9	1.6
Traes_6AS_9557563D1	lipoxygenase	1.8	1.3
Traes_5DL_5CF73B088	DREB transcription factor 5B (DREB5)	1.2	1.3
Traes_4AS_773C02521	1-aminocyclopropane-1-carboxylate oxidase-like	2.4	3.2
Traes_4BL_CF12F5942 (Q11)	1-aminocyclopropane-1-carboxylate oxidase-like	1.6	1.9
Novel08424 (Q12)	putative ethylene-responsive transcription factor	1.6	Inf
Novel04144 (Q13)	TaAP2-D	1.9	Inf
Novel02151 (Q14)	ABI3-interacting protein 2–2	1.6	1.4
Traes_2AL_A26170C43 (Q15)	cytokinin response regulator 2 (crr2)	1.1	1.4
Traes_4AL_F4C83730F	cytokinin riboside 5′-monophosphate phosphoribohydrolase	1.0	1.3
**Transcription factor and signaling regulation**			
Novel00797 (Q16)	MADS-box transcription factor 23-like	3.5	1.9
Traes_4DL_D73F1E523 (Q17)	zinc finger protein 2-like	2.3	1.3
Traes_2DL_7D58E9850 (Q18)	B-box zinc finger protein 20-like	1.3	1.0
Traes_5DL_B2F166A31	TaMYB61 MYB-related protein	1.1	1.3
Traes_7AL_6E1780317	L10-interacting MYB domain-containing protein-like	1.0	Inf
Novel07179	putative Zn-finger protein	1.1	Inf
Traes_4BL_78DD63002	homeobox-leucine zipper protein HOX19	1.2	1.4
Traes_4BS_47C0E033A	CBL-interacting protein kinase 14	2.0	1.3
Traes_4AL_A8EEBE537	CBL-interacting protein kinase 14 (CIPK14)	2.9	1.8
Traes_4DS_DBAA2CC451	CBL-interacting protein kinase 14 (CIPK14)	1.8	1.6

These candidate genes were up-regulated in both the S_ *st1* vs *st1* and S_ *st1* vs S_WT comparison groups with the corrected *p* value < 0.05 and are here classified according to predicted gene function. Q1–Q18 in brackets indicates gene expression was confirmed by qPCR; ^a^The values in the columns are the log2 Fold Change values for the S_ *st1* vs *st1* or S_ *st1* vs S_WT comparison groups.

Among genes involved in arginine and proline metabolism, transcript levels of five genes encoding polyamine oxidase (PAO) family members were up-regulated in the S_*st1* vs *st1* and the S_*st1* vs S_WT comparisons (Table 2). Similar expression patterns for these genes were observed in qPCR analysis (Fig. 6, Q1–Q4). The mRNA of the gene encoding arginine decarboxylase (ADC), an enzyme that catalyzes the synthesis of Put, was also detected as having increased expression in the S_*st1* vs *st1* and the S_*st1* vs S_WT comparisons (Table 2). PAO and ADC genes are critical for PA metabolism, and PAs play important roles in plant resistance to environmental stresses^11, 14, 46–48^. For example, it has been reported that a cotton PAO is required for the mediation of spermine and camalexin signaling to resist a destructive fungal pathogen^16^. Additionally, PAO has also been reported to contribute to improving drought stress tolerance in grapevine^15^. It has been shown that Put plays a positive role in salt tolerance by attenuating oxidative damage in soybean roots^12^. Furthermore, by modulating nutrient acquisition and the PA pool, inoculation with arbuscular mycorrhizal fungi improves adaptation to saline soils^49^. Several studies have suggested that the modulation of PA catabolism is important for salinity tolerance^50–53^. In this study, the expression levels of key genes in PA metabolism were increased in the S_*st1* vs *st1* and the S_*st1* vs S_WT comparisons, suggesting that the modulation of PAs is important for salt tolerance in *st1*.

Oxidation-reduction processes are also critical for salinity tolerance in plants. In accordance with the enrichment of the GO term “oxidation-reduction process” in up-regulated genes of the S_*st1* vs S_WT comparison, several oxidoreductase genes, including genes encoding cytochrome P450 monooxygenases (CYP), ascorbate oxidoreductase and glutaredoxin, had increased expression in the S_*st1* vs *st1* and the S_*st1* vs S_WT comparisons. Specifically, eleven genes putatively encoding CYP were up-regulated in the S_*st1* vs *st1* and the S_*st1* vs S_WT comparisons (Table 2). Similar expression patterns for these genes were observed in qPCR analysis. (Fig. 6, Q5–Q9). CYPs catalyze the biooxidation of various substrates through activation of molecular oxygen and play important roles in metabolic processes and stress responses^54^. It has been suggested that increased expression of *CYP94* genes in rice plants alleviates jasmonate responses and enhances salt tolerance^55^. Mutation of CYP709B3 in *Arabidopsis* conferred sensitivity to ABA and low tolerance to salt stress, indicating that CYP709B3 plays a role in ABA responses and salinity tolerance^56^. In addition, two genes encoding L-ascorbate oxidase-like and ascorbate-dependent oxidoreductase, which are involved in ascorbate recycling, had increased expression in the S_*st1* vs *st1* and the S_*st1* vs S_WT comparisons (Table 2). Ascorbate plays important roles in protecting plants against various stresses by regulating cellular H_2_O_2_ levels^57^. Two glutaredoxin-C1 genes, the glutathione-dependent enzyme involved in reducing disulfide bridges, were up-regulated in the S_*st1* vs *st1* and the S_*st1* vs S_WT comparisons (Table 2). Overexpression of tomato glutaredoxin results in tolerance to oxidative, drought, and salt stresses, suggesting that glutaredoxin plays a crucial role in regulating abiotic tolerance^58^. On the whole, the oxidoreductases are involved in scavenging reactive oxygen species and maintaining oxidation-reduction homeostasis^3^ and thus protect the salt-treated plants from damage.

Hormones, such as jasmonate, ethylene, abscisic acid and cytokinins, are also known to be involved in the regulation of salinity tolerance in plants^59^. Increased expression of allene oxide cyclase in wheat and *Arabidopsis* resulted in increased jasmonate content and in improved resistance to salinity^60^. In our study, allene oxide synthase, a key gene involved in the biosynthesis of jasmonic acid, was highly expressed in salt-treated *st1* (Table 2). qPCR analysis also showed that this gene had significantly higher expression in salt-treated *st1* than in untreated *st1* or in salt-treated WT (Fig. 6, Q10). Two 12-oxophytodienoate reductase genes and four lipoxygenase family genes, which also participate in the jasmonate synthesis pathway, and had increased expression levels in the S_*st1* vs *st1* and the S_*st1* vs S_WT comparisons (Table 2). The high expression of these jasmonate biosynthetic genes may contribute to salinity tolerance in *st1* under high salinity. Makhlouf *et al*. reported that an ethylene response factor gene is probably associated with salt tolerance in wheat^61^; ethylene also plays roles in salt responses in plants^59, 62, 63^. Five genes involved in ethylene biosynthesis or regulation, were up-regulated in the S_*st1* vs *st1* and the S_*st1* vs S_WT comparisons (Table 2), and these expression patterns were confirmed by qPCR (Fig. 6, Q11–Q13). Changes in the expression of ethylene-related genes observed in the S_*st1* vs *st1* and the S_*st1* vs S_WT comparisons indicate that ethylene may also contribute to salinity tolerance in *st1*. Interestingly, the expression of putative ethylene-responsive transcription factor (Q12) and TaAP2-D (Q13) genes was barely detectable in WT and salinity-treated WT but highly expressed in *st1* (Fig. 6). The two novel genes may be involved in salinity tolerance in the *st1* mutant plants. In addition, a transcription factor involved in abscisic acid signal transduction and two genes related to cytokinin showed increased expression levels in the S_*st1* vs *st1* and the S_*st1* vs S_WT comparisons (Table 2 and Fig. 6, Q14–Q15). Taken together, the high expression of genes related to jasmonate, ethylene, abscisic acid, and cytokinins in the salt-treated *st1* mutant suggest that these hormones may explain the improved tolerance to salt stress observed in this mutant.

More than ten transcription factor and signal transduction genes were up-regulated in the S_*st1* vs *st1* and the S_*st1* vs S_WT comparisons (Table 2). Similar expression patterns were observed for the selected genes in qPCR analysis. (Fig. 6, Q16–Q18). It has been reported that high levels of expression of the Na^+^-induced *TaMYB73* gene in *Arabidopsis* results in increased tolerance to salt stress^64^. In our study, two salt-induced genes encoding MYB-related proteins were more highly expressed in salt-treated *st1* compared with salt-treated WT plants (Table 2), suggesting that this gene may function in tolerance to salt stress in *st1*. Three genes encoding CBL-interacting protein kinases (CIPK) were up-regulated in the S_*st1* vs *st1* and the S_*st1* vs S_WT comparisons (Table 2). The CBL/CIPK network has been shown to regulate the Na^+^ efflux transporter SOS1 in *Arabidopsis* ^18, 23, 65^. Several CIPK genes from various plants have been reported to function in salt tolerance^21–23^, and it is reasonable to speculate that the high levels of expression of CIPK genes in *st1* promotes tolerance to salt stress. Additionally, some transcription factors, including a MADS-box gene (Fig. 6, Q16), zinc finger proteins (Fig. 6, Q17–18), and a homeobox-leucine zipper protein, were newly identified as putative salt tolerance genes in this study (Table 2 and Fig. 6).

In conclusion, comparative analysis of the shoot transcriptomes of WT and the space-induced wheat mutant *st1* not only indicates that multiple genes and pathways are related to plant responses to salinity stress, but also provides a catalog of transcriptomic sequence variation induced by spaceflight. The data generated in this study will be a valuable resource for the identification of key genes related to salinity tolerance in wheat and also for studies of the sequence variation resulting from induced mutation.

## Materials and Methods

### Plant materials

The core parent Jimai 20 from Shandong province was used as the WT, and mutation was induced by spaceflight on the Shijian-8 satellite in 2006. The satellite Shijian-8 was in orbit for 15 days; its mission was specific to seed-breeding programs. The average irradiation dose for the seeds in the cabin of the satellite was about 2.894 mGy, and the mean gravity was 1. 3 × 10^−3^ g^66^. The M_6_ generation was used for salt-tolerance screening. Salt-tolerance screening was conducted in 1.5% NaCl solution for several days, and then the growth of the mutants was monitored. One mutant, named *st1*, with a high germination rate and superior growth characteristics was selected for further analysis.

### Salt treatment and physiological analysis

After germination for 2–3 days in deionized water, the WT and mutant were grown in half concentration nutrient solution without NaCl, or with 100, 200, 300 mM NaCl for five days under 200–300 µmol m^−2^ s^−1^ light at 21 °C in a growth chamber. The composition of nutrient solution was according to Guo *et al*.^67^. The shoot weights from five independent replicates of WT and *st1* plants treated with different concentrations of NaCl were measured. The Na^+^ content in the shoot was measured from at least three replicates according to a previously-published method^2^. The malondialdehyde (MDA) content was measured as described previously^68^. For germination analysis, the WT and *st1* seeds were directly cultured in water containing 250 mM NaCl for several days. The germination rate was calculated as the number of seeds germinated divided by the total number of seed.

### RNA extraction and library construction

The shoots from five plants for each treatment were pooled as one sample replicate. Two replicates for each treatment were prepared to isolate total RNA. Total RNA was isolated by using an RNeasy Plant Mini Kit (Qiagen) according to the product instructions. DNase I (Takara) and an RNA purification kit (Tiangen) were used to eliminate DNA contamination. The purity, concentration and integrity of RNA was assessed by using a NanoPhotometer® spectrophotometer (IMPLEN), a Qubit® RNA Assay Kit with a Qubit® 2.0 Flurometer (Life Technologies), and an RNA Nano 6000 Assay Kit with the Bioanalyzer 2100 system (Agilent Technologies). Sequencing libraries were generated using an NEBNext® Ultra™ RNA Library Prep Kit for Illumina® (NEB) following the manufacturer’s recommendations. Briefly, mRNA was purified by using poly-T oligo-attached magnetic beads. After fragmentation, the cDNA was synthesized, and NEBNext Adaptors with hairpin loop structures were ligated to prepare for hybridization. PCR products were purified (AMPure XP system), and library quality was assessed on an Agilent Bioanalyzer 2100 system. Finally, eight libraries were successfully constructed.

### Transcriptome analysis

The libraries were deep sequenced with the Illumina Hiseq platform. For sequence quality control, clean reads were obtained by removing reads containing adapters, reads containing poly-N homopolymers and other low quality reads from the raw data. The Q20, Q30, and GC content in the clean reads were calculated to further assess quality. Release-31 version of the wheat reference genome and gene model annotation files were downloaded from the genome website (ftp://ftp.ensemblgenomes.org/pub/plants/release-31/fasta/triticum_aestivum/dna/). An index of the reference genome was then built using Bowtie v2.2.3, and paired-end clean reads were aligned to the reference genome using TopHat v2.0.12. For SNP analysis, Picard-tools v1.96 and samtools v0.1.18 were used to sort, mark duplicated reads, and reorder the bam alignment results of each sample. GATK2 (v3.2) software was used to perform SNP calling.

### Quantification of gene expression levels and DEG analysis

The number of reads mapped to each gene were counted using HTSeq v0.6.1. The expected number of Fragments Per Kilobase of transcript sequence per Millions base pairs sequenced (FPKM) of each gene was then calculated based on the length of the gene and the read count mapped to this gene. FPKM, reflecting both the effect of sequencing depth and gene length for the read count, is a commonly used method for estimating gene expression levels^69^. DEG analysis was performed using the DESeq R package (1.18.0). DESeq provides statistical routines for determining DEGs using a model based on a negative binomial distribution. The resulting *P*-values were adjusted using the Benjamini and Hochberg approach for controlling the false discovery rate. Genes with an adjusted *P*-value determined to be <0.05 (FDR < 0.05) by DESeq and that had a fold change value ≥2 (|Log2 fold change| ≥ 1) between two groups were considered to be differentially expressed.

### GO and KEGG enrichment analysis

For gene ontology (GO) mapping, the GO terms of DEGs associated with homologies (GO; http://www.geneontology.org) were extracted. GO enrichment analysis of DEGs was implemented using the GOseq R package, in which gene length bias was corrected. GO terms with corrected *P* values less than 0.05 were considered to be significantly enriched. REVIGO was used for analysis of the enriched GO terms (http://revigo.irb.hr/); this program removes redundant GO terms and attempts to reflect the similarity of given terms by semantic space^34^. The ten biological process category GO terms with the lowest *p* values for enrichment in both the up- or down-regulated genes in the S_*st1* vs S_WT comparisons were analyzed by REVIGO. KEGG pathways for the DEGs were retrieved (http://www.genome.jp/kegg/), and KOBAS software was used to test the statistical significance of the enrichment of DEGs in KEGG pathways.

### Quantitative real-time PCR

The shoots from salt-treated and control wheat plants were sampled. Five plants were mixed as one replicate, and three independent biological replicates with three technical replicates of each biological replicate were analyzed. Total RNA was isolated using an RNeasy Plant Mini Kit (Qiagen). DNA contamination was removed using DNase I (Takara) and an RNA purification kit (Tiangen). First-strand cDNA was synthesized using an iScript cDNA synthesis kit (Bio-Rad). Quantitative real-time PCR was conducted by using SsoFast EvaGreen Supermix Kit (Bio-Rad) on a CFX 96 Real-Time System (Bio-Rad). The primers used for quantitative real-time PCR are detailed in Supplementary Table S7.

## Electronic supplementary material

Dataset 1

Dataset 2

Dataset 3

Dataset 4

Dataset 5

Dataset 6

Dataset 7

## References

[1] Hauser F, Horie T (2010). A conserved primary salt tolerance mechanism mediated by HKT transporters: a mechanism for sodium exclusion and maintenance of high K(+)/Na(+) ratio in leaves during salinity stress. Plant Cell Environ..

[2] Guan R (2014). Salinity tolerance in soybean is modulated by natural variation in GmSALT3. Plant J..

[3] Parihar P, Singh S, Singh R, Singh VP, Prasad SM (2015). Effect of salinity stress on plants and its tolerance strategies: a review. Environ. Sci. Pollut. R..

[4] Rahnama A, Munns R, Poustini K, Watt M (2011). A screening method to identify genetic variation in root growth response to a salinity gradient. J. Exp. Bot..

[5] Ling HQ (2013). Draft genome of the wheat A-genome progenitor *Triticum urartu*. Nature.

[6] Bouthour D, Kalai T, Chaffei HC, Gouia H, Corpas FJ (2015). Differential response of NADP-dehydrogenases and carbon metabolism in leaves and roots of two durum wheat (*Triticum durum* Desf.) cultivars (Karim and Azizi) with different sensitivities to salt stress. J. Plant Physiol..

[7] Colmer TD, Flowers TJ, Munns R (2006). Use of wild relatives to improve salt tolerance in wheat. J. Exp. Bot..

[8] Astolfi S, Zuchi S (2013). Adequate S supply protects barley plants from adverse effects of salinity stress by increasing thiol contents. Acta. Physiol. Plant.

[9] Fatma M, Masood A, Per TS, Rasheed F, Khan NA (2016). Interplay between nitric oxide and sulfur assimilation in salt tolerance in plants. Crop J..

[10] Chen M, Chen JJ, Fang JY, Guo ZF, Lu SY (2014). Down-regulation of S-adenosylmethionine decarboxylase genes results in reduced plant length, pollen viability, and abiotic stress tolerance. Plant Cell Tiss. Org..

[11] Gupta K, Dey A, Gupta B (2013). Plant polyamines in abiotic stress responses. Acta. Physiol. Plant.

[12] Zhang GW, Xu SC, Hu QZ, Mao WH, Gong YM (2014). Putrescine plays a positive role in salt-tolerance mechanisms by reducing oxidative damage in roots of vegetable soybean. J. Integr. Agr.

[13] Zhao FG, Sun C, Liu YL, Zhang WH (2003). Relationship between polyamine metabolism in roots and salt tolerance of barley seedlings. Acta. Bot. Sin..

[14] Rangan, P., Subramani, R., Kumar, R., Singh, A. K. & Singh, R. Recent advances in polyamine metabolism and abiotic stress tolerance. *Biomed*. *Res*. *Int* (2014). 10.1155/2014/239621 PMC4124767 25136565

[15] Hatmi S (2015). Drought stress tolerance in grapevine involves activation of polyamine oxidation contributing to improved immune response and low susceptibility to Botrytis cinerea. J. Exp. Bot..

[16] Mo HJ (2015). Cotton polyamine oxidase is required for spermine and camalexin signalling in the defence response to Verticillium dahliae. Plant J..

[17] Shi H, Ishitani M, Kim C, Zhu JK (2000). The *Arabidopsis thaliana* salt tolerance gene SOS1 encodes a putative Na^+^/H^+^ antiporter. Proc. Natl. Acad. Sci..

[18] Kudla J, Batistic O, Hashimoto K (2010). Calcium signals: the lead currency of plant information processing. Plant Cell.

[19] Liu J, Ishitani M, Halfter U, Kim CS, Zhu JK (2000). The *Arabidopsis thaliana* SOS2 gene encodes a protein kinase that is required for salt tolerance. Proc. Natl. Acad. Sci..

[20] Mahajan S, Pandey GK, Tuteja N (2008). Calcium- and salt-stress signaling in plants: shedding light on SOS pathway. Arch. Biochem. Biophys..

[21] Zhang H, Lv F, Han X, Xia X, Yin W (2013). The calcium sensor PeCBL1, interacting with PeCIPK24/25 and PeCIPK26, regulates Na^+^/K^+^ homeostasis in *Populus euphratica*. Plant Cell Rep..

[22] Chen X (2014). ZmCIPK21, a maize CBL-interacting kinase, enhances salt stress tolerance in *Arabidopsis thaliana*. Int. J. Mol. Sci..

[23] Li R (2012). HbCIPK2, a novel CBL-interacting protein kinase from halophyte Hordeum brevisubulatum, confers salt and osmotic stress tolerance. Plant Cell Environ.

[24] Buyuk I (2016). Genome-wide identification of salinity responsive HSP70s in common bean. Mol. Biol. Rep..

[25] Bushman BS, Amundsen KL, Warnke SE, Robins JG, Johnson PG (2016). Transcriptome profiling of Kentucky bluegrass (*Poa pratensis* L.) accessions in response to salt stress. BMC Genomics.

[26] Tsukagoshi H (2015). RNA-Seq analysis of the response of the halophyte, *Mesembryanthemum crystallinum* (ice plant) to high salinity. PLoS One.

[27] Long WH, Zou XL, Zhang XK (2015). Transcriptome analysis of canola (*Brassica napus*) under salt stress at the germination stage. PLoS One.

[28] Zhang J (2014). Transcriptome dynamics of a desert poplar (*Populus pruinosa*) in response to continuous salinity stress. Plant Cell Rep..

[29] Goyal E (2016). Transcriptome profiling of the salt-stress response in *Triticum aestivum* cv. Kharchia Local. Sci. Rep..

[30] Pathirana R (2011). Plant mutation breeding in agriculture. CAB Rev..

[31] Levinskikh MA (2000). Analysis of the spaceflight effects on growth and development of super dwarf wheat grown on the space station Mir. J. Plant Physiol..

[32] Visscher AM (2009). Effects of a spaceflight environment on heritable changes in wheat gene expression. Astrobiology.

[33] Paul AL (2012). Spaceflight transcriptomes: unique responses to a novel environment. Astrobiology.

[34] Svenja Fengler IS, Neef M, Ecke M, Nieselt K (2015). Rüdiger Hampp. A whole-genome microarray study of *Arabidopsis thaliana* semisolid callus cultures exposed to microgravity and nonmicrogravity related spaceflight conditions for 5 days on board of Shenzhou 8. Biomed Res. Int..

[35] Sugimoto M (2014). Genome-wide expression analysis of reactive oxygen species gene network in Mizuna plants grown in long-term spaceflight. BMC Plant Biol..

[36] Paul AL, Zupanska AK, Schultz ER, Ferl RJ (2013). Organ-specific remodeling of the *Arabidopsis* transcriptome in response to spaceflight. BMC Plant Biol..

[37] Peng H (2015). Transcriptomic changes during maize roots development responsive to Cadmium (Cd) pollution using comparative RNAseq-based approach. Biochem. Biophys. Res. Commun..

[38] Gan, L. *et al*. *De Novo* transcriptome analysis for Kentucky bluegrass dwarf mutants induced by space mutation. *Plos One* **11** (2016). 10.1371/journal.pone.0151768 PMC4807101 27010560

[39] Li GT (2016). Genome-wide sequencing of 41 rice (*Oryza sativa* L.) mutated lines reveals diverse mutations induced by fast-neutron irradiation. Mol. Plant.

[40] Shirasawa K, Hirakawa H, Nunome T, Tabata S, Isobe S (2016). Genome-wide survey of artificial mutations induced by ethyl methanesulfonate and gamma rays in tomato. Plant Biotechnol. J..

[41] Supek F, Bosnjak M, Skunca N, Smuc T (2011). REVIGO summarizes and visualizes long lists of gene ontology terms. PLoS One.

[42] Munns R (2012). Wheat grain yield on saline soils is improved by an ancestral Na^+^ transporter gene. Nat. Biotechnol..

[43] Byrt CS (2014). The Na^+^ transporter, TaHKT1;5-D, limits shoot Na^+^ accumulation in bread wheat. Plant J..

[44] Khan, H. A., Siddique, K. H. M. & Colmer, T. D. Vegetative and reproductive growth of salt-stressed chickpea are carbon-limited: sucrose infusion at the reproductive stage improves salt tolerance. *J*. *Exp*. *Bot*. doi:10.1093/jxb/erw177 (2016). 10.1093/jxb/erw177 PMC5429013 27140441

[45] Zheng L (2015). Transcriptomic analysis reveals importance of ROS and phytohormones in response to short-term salinity stress in *Populus tomentosa*. Front Plant Sci..

[46] Hussain SS, Ali M, Ahmad M, Siddique KHM (2011). Polyamines: Natural and engineered abiotic and biotic stress tolerance in plants. Biotechnol. Adv..

[47] Jimenez-Bremont JF (2014). Physiological and molecular implications of plant polyamine metabolism during biotic interactions. Front Plant Sci..

[48] Wimalasekera R, Tebartz F, Scherer GFE (2011). Polyamines, polyamine oxidases and nitric oxide in development, abiotic and biotic stresses. Plant Sci..

[49] Talaat NB, Shawky BT (2013). Modulation of nutrient acquisition and polyamine pool in salt-stressed wheat (*Triticum aestivum* L.) plants inoculated with arbuscular mycorrhizal fungi. Acta. Physiol. Plant.

[50] Parvin S (2012). Modulation of polyamine levels in ginseng hairy root cultures subjected to salt stress. Russ. J. Plant Physiol..

[51] Piterkova J, Luhova L, Zajoncova L, Sebela M, Petrivalsky M (2012). Modulation of polyamine catabolism in pea seedlings by calcium during salinity stress. Plant Protect. Sci..

[52] Qing DJ (2009). Comparative profiles of gene expression in leaves and roots of maize seedlings under conditions of salt stress and the removal of salt stress. Plant Cell Physiol..

[53] Radyukina NL (2009). Homeostasis of polyamines and antioxidant systems in roots and leaves of Plantago major under salt stress. Russ. J. Plant Physiol..

[54] Werckreichhart D (2000). Cytochromes P450: a success story. Genome Biol..

[55] Kurotani K (2015). Elevated levels of CYP94 family gene expression alleviate the jasmonate response and enhance salt tolerance in rice. Plant Cell Physiol.

[56] Mao GH, Seebeck T, Schrenker D, Yu O (2013). CYP709B3, a cytochrome P450 monooxygenase gene involved in salt tolerance in *Arabidopsis thaliana*. BMC Plant Biol..

[57] Ishikawa T, Shigeoka S (2008). Recent advances in ascorbate biosynthesis and the physiological significance of ascorbate peroxidase in photosynthesizing organisms. Biosci. Biotechnol. Biochem..

[58] Guo YS, Huang CJ, Xie Y, Song FM, Zhou XP (2010). A tomato glutaredoxin gene SlGRX1 regulates plant responses to oxidative, drought and salt stresses. Planta.

[59] Ryu H, Cho YG (2015). Plant hormones in salt stress tolerance. J. Plant Biol..

[60] Zhao Y (2014). A wheat allene oxide cyclase gene enhances salinity tolerance via jasmonate signaling. Plant Physiol..

[61] Makhloufi E (2014). Isolation and molecular characterization of ERF1, an ethylene response factor gene from durum wheat (*Triticum turgidum* L. subsp. durum), potentially involved in salt-stress responses. J. Exp. Bot..

[62] Cao YR, Chen SY, Zhang JS (2008). Ethylene signaling regulates salt stress response: An overview. Plant Signal Behav..

[63] Cao WH (2006). Expression of tobacco ethylene receptor NTHK1 alters plant responses to salt stress. Plant Cell Environ..

[64] He Y (2012). Ectopic expression of a wheat MYB transcription factor gene, TaMYB73, improves salinity stress tolerance in *Arabidopsis thaliana*. J. Exp. Bot..

[65] Quan R (2007). SCABP8/CBL10, a putative calcium sensor, interacts with the protein kinase SOS2 to protect *Arabidopsis* shoots from salt stress. Plant Cell.

[66] Guo HJ (2010). Mutagenic effects of different factors in spaceflight environment of Shijian-8 satellite in wheat. Acta Agronomica Sinica.

[67] Guo Y (2012). QTL mapping for seedling traits in wheat grown under varying concentrations of N, P and K nutrients. Theor. Appl. Genet..

[68] Draper HH, Hadley M (1990). Malondialdehyde determination as index of lipid peroxidation. Methods Enzymol..

[69] Trapnell C (2010). Transcript assembly and quantification by RNA-Seq reveals unannotated transcripts and isoform switching during cell differentiation. Nat. Biotechnol..

