# Supplementary material for: RNAseq analysis reveals pathways and candidate genes associated with salinity tolerance in a spaceflight-induced wheat mutant
Source: Sci Rep. 2017 Jun 2;7:2731. doi: 10.1038/s41598-017-03024-0 (PMC5457441; doi:10.1038/s41598-017-03024-0)
Supplement: Supplementary file 1 — Dataset 1 [file 41598_2017_3024_MOESM1_ESM.pdf]

Supplementary information

RNAseq analysis reveals pathways and candidate genes associated with salinity tolerance in a spaceflight-induced wheat mutant

Hongchun Xiong, Huijun Guo, Yongdun Xie, Linshu Zhao, Jiayu Gu, Shirong Zhao, Junhui Li & Luxiang Liu*

Institute of Crop Science, Chinese Academy of Agricultural Sciences/National Key Facility for Crop Gene Resources and Genetic Improvement, National Center of Space Mutagenesis for Crop Improvement, Beijing 100081, China

•Corresponding author. E-mail address: liuluxiang@caas.cn;
Tel: +86 10 62122719

Table S1 Summary of the sequencing data generated for WT and mutant wheat transcriptome and quality filtering.

Sample name	Raw reads	Clean reads	Total mapped (%)	Uniquely mapped (%)	Multiple mapped (%)	Q20 (%)	Q30 (%)	GC content (%)
WT_1	55304346	53620402	67.54	58.63	8.91	98.22	95.70	59.79
WT_2	43780896	42412680	67.90	59.47	8.43	98.22	95.72	59.08
*st1*_1	50064754	48468954	68.59	59.62	8.97	98.24	95.76	59.57
*st1*_2	59493856	57650410	69.22	60.53	8.69	98.19	95.65	59.94
S_WT_1	57701510	55847070	68.67	60.28	8.39	98.18	95.63	59.25
S_WT_2	65439896	63451116	68.22	59.88	8.34	98.24	95.76	58.41
S_*st1*_1	61690128	59752716	68.57	60.04	8.53	98.37	96.01	58.65
S_*st1*_2	54837928	53139340	68.38	59.88	8.49	98.40	96.07	58.83

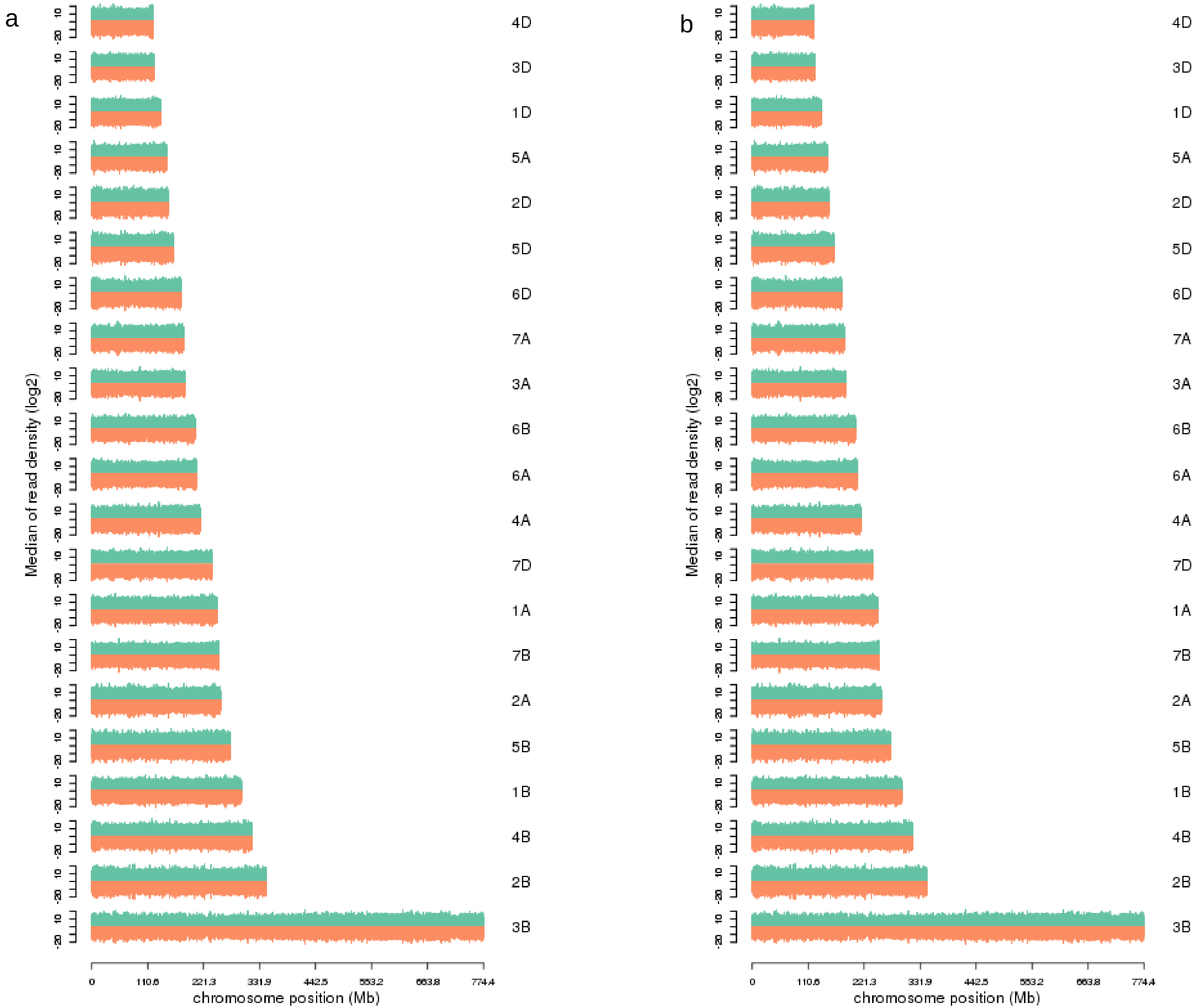

Figure S1 Reads density in chromosomes. (a) WT_1; (b) *st1*_1. The chromosome position and length was according to the wheat reference genome. The ordinate scales indicate log2 of the median of reads mapped to each chromosome of the wheat genome. Green and red colors indicate the positive and negative strands, respectively.

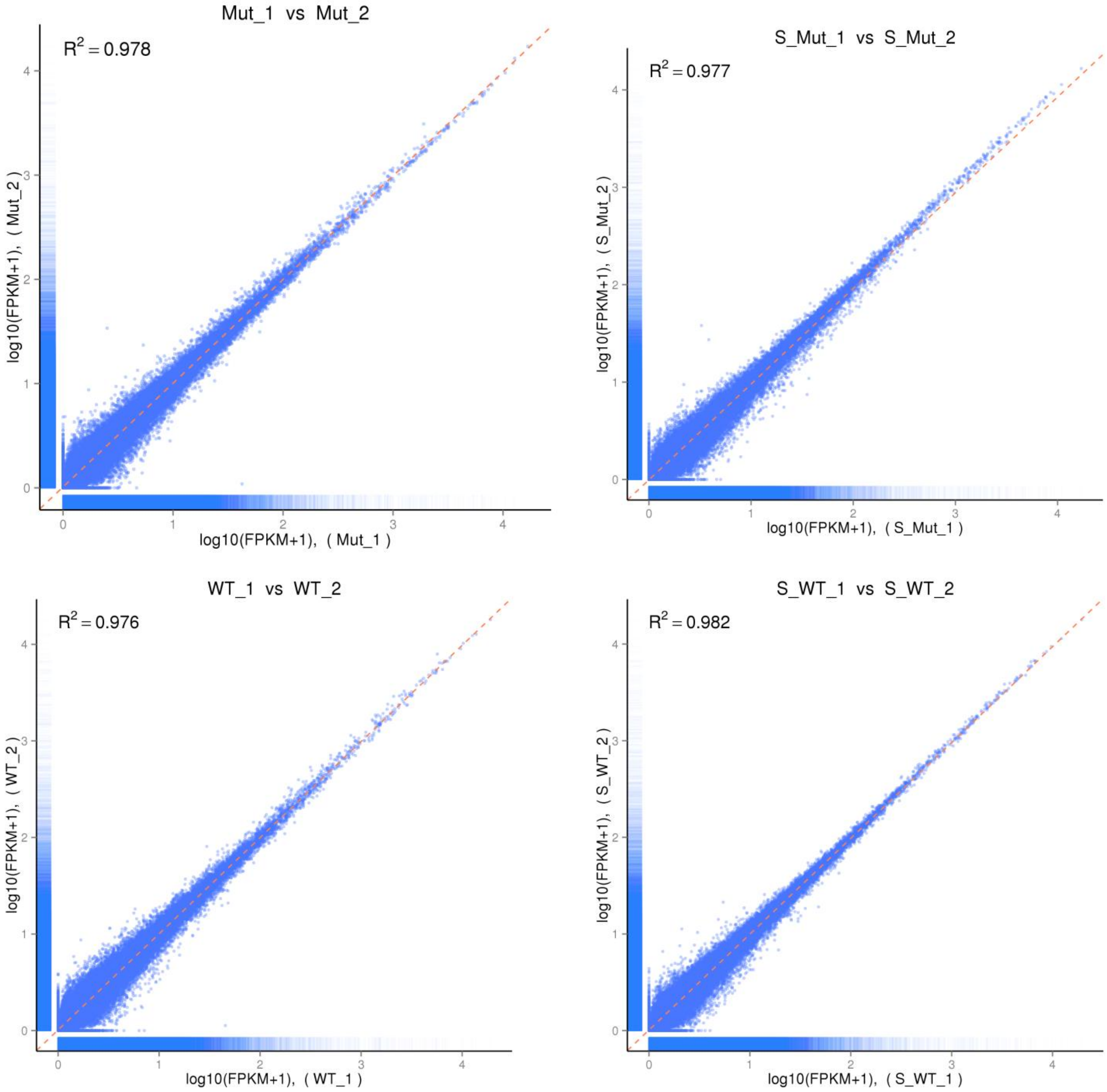

Figure S2 Linear relationship of the expression levels between two biological replicates. The indications in the top of the figures are sample names and R^2 is the correlation coefficient.

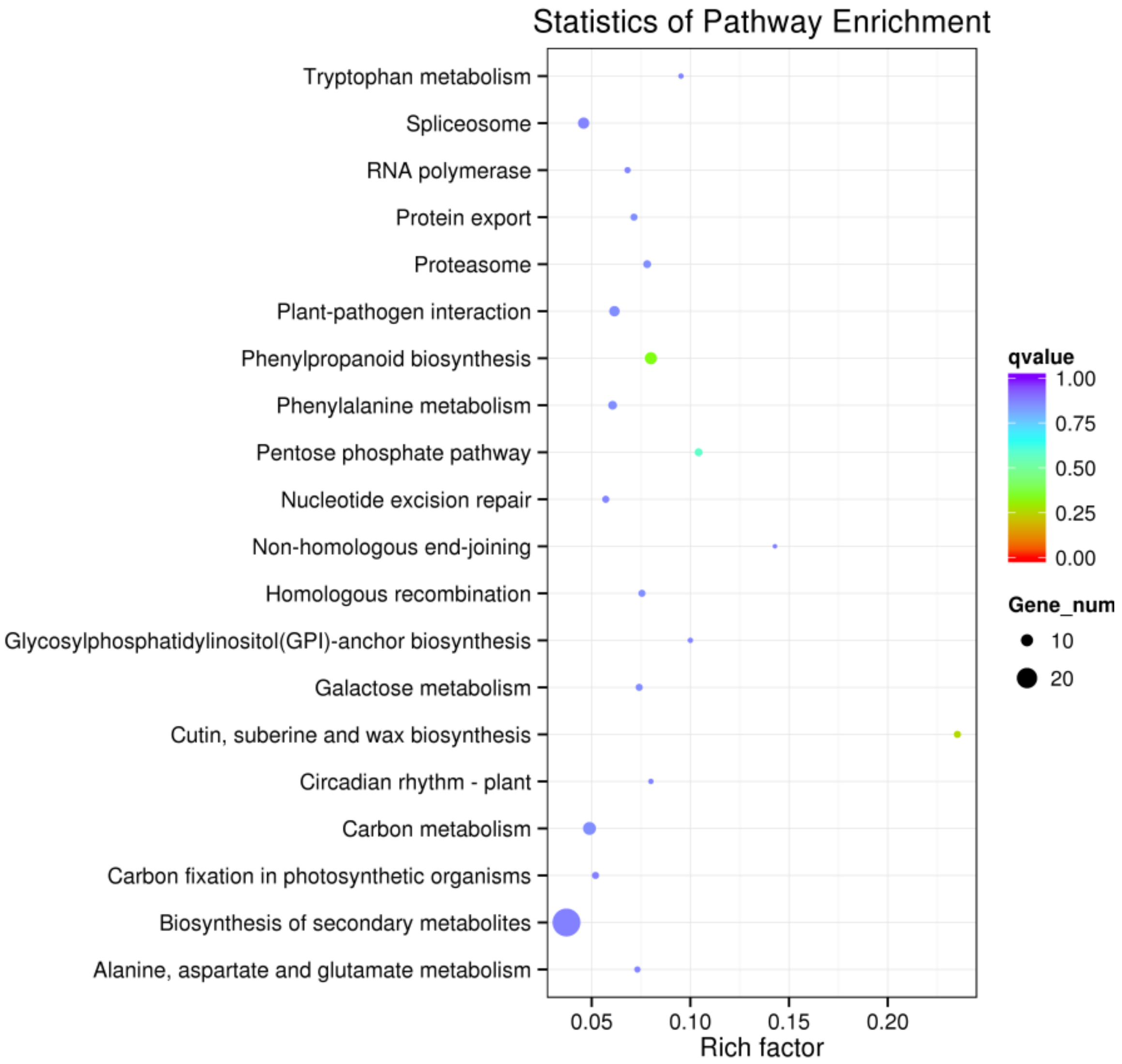

Figure S3 KEGG pathways enriched by down-regulated genes in the S_*st1* vs S_WT. The abscissa represents rich factor reflecting the proportion of DEGs in the pathway. The number of DEGs in the pathway was indicated by the circle area and the circle color represents the corrected *P* value.
